# Efficacy and Tolerance of IMRT Boost Compared to IORT Boost in Early Breast Cancer: A German Monocenter Study

**DOI:** 10.3390/cancers14246196

**Published:** 2022-12-15

**Authors:** Luisa Schumacher, Joke Tio, Hans Theodor Eich, Gabriele Reinartz

**Affiliations:** 1 Department of Radiation Oncology, University Hospital of Muenster, 48149 Muenster, Germany; 2 Department of Obstetrics and Gynecology, Breast Center, University Hospital of Muenster, 48149 Muenster, Germany

**Keywords:** breast cancer, IORT, intraoperative radiotherapy, IMRT, intensity-modulated radiotherapy, boost, cosmesis, acute toxicity, late toxicity

## Abstract

**Simple Summary:**

The benefit of administering an extra dose to the tumor bed (boost) prior or simultaneously to adjuvant breast radiotherapy in terms of local recurrence rate has already been the subject of several studies and, thus, has already found justification in appropriate patient cases. In this study, we compared two boost techniques, an intraoperative radiotherapy (IORT) boost and an intensity-modulated radiotherapy boost (IMRT), analyzing two patient collectives to determine the equivalence of the two techniques or which of the two proves superior. According to our monocenter data on tumor control, survival rates, toxicities, and cosmetic results, no significant differences were revealed between the two boost techniques, and it cannot be generalized that one technique is more advantageous than the other.

**Abstract:**

The aim of this retrospective study is to compare the two boost subgroups, IORT or IMRT, in terms of overall survival (OS), progression-free survival (PFS), cosmesis, and acute and late toxicity. It shall be shown whether and which of the boost techniques offers better results with respect to the facial points, since there are already many studies on applying boost to the tumor bed after/during breast conserving surgery, and there are few which compare the different techniques. For this comparison, two subgroups of 76 patients each (*n* = 152), treated between 2002 and 2015, were enrolled in the study. In one subgroup, the 9 Gy boost was intraoperatively administered after complete removal of the primary tumor, while the other subgroup received the boost of 8.4 Gy percutaneously and simultaneously integrated into the tumor bed after breast conserving surgery. Both subgroups have subsequently undergone whole breast irradiation (WBI) of 50.4/50 Gy in 1.8–2 Gy per fraction. OS and the incidence of late toxicity did not differ between the two subgroups and no risk factor was found regarding PFS. Acute toxicities initially occurred significantly less (*p* < 0.001) in the IORT subgroup; however, after WBI took place, this difference vanished. Therefore, boost application by means of IORT or IMRT can be considered equivalent.

## 1. Introduction

Whole breast irradiation after lumpectomy and sentinel lymph node biopsy is the standard therapy for invasive breast cancer. The current guidelines specify that radiation can be administered either hypofractionated, with 40 Gy in 15–16 fractions, or normofractionated, with 50 Gy in 25–28 fractions [1]. In some cases, the radiotherapy can be omitted [2,3]. However, the current state of research reports that there is no group that does not benefit from radiation in terms of decreasing recurrence rates [4,5,6,7,8,9]. Since local recurrences are most likely to occur in the same quadrant of primary lesion, the idea of a local dose saturation (boost) has been justified [10,11]. A boost to the tumor bed can be applied by external beam treatment or brachytherapy. Over time, the techniques for intraoperative radiotherapy (IORT) have been revised. IORT, as a boost prior to whole breast irradiation, includes various techniques and dosage concepts, all of which should be considered differently; however, the concept itself promises many benefits [12,13,14]. Intraoperative radiotherapy prolongs surgical time but shortens post-operative radiotherapy [15,16]. Whether IORT can function as a sole adjuvant radiotherapy without whole breast irradiation is the subject of ongoing studies [17,18,19,20]. Since the skin as a critical organ for late complications is spared, a better cosmetic result and less late toxicity are hoped for and, thus, IORT seems to be a comparable alternative to conventional postoperative fractionated boost radiotherapy [12,21]. In this present study, the aforementioned benefits of IORT are compared to the administration of intensity-modulated radiotherapy (IMRT) boosts on various aspects, as the data situation is still small on this topic. A randomized study by Ciabattoni et al., published in 2021, comparing intraoperative radiotherapy with electrons (IOERT) versus external beam electron boost appears to be the only randomized trial that compares these boost applications [22]. In this study, due to the lack of information, no findings on modern prognostic factors such as Her2 status can be made. Another sequential intervention study by Reitsamer et al., focusing on the local recurrence rate in patients treated with intraoperative-electron boost radiotherapy versus post-operation external beam electron boost irradiation, published in 2004, explains initial conclusions on local recurrence rate, late and acute toxicity, and cosmesis, with the conclusion that further evaluation is needed to support the theses [23]. This present study serves to examine these theses and to draw a comparison between the two boost applications.

## 2. Materials and Methods

This German monocenter retrospective case study evaluated adjuvant radiotherapy administrated intraoperative radiotherapy (IORT) versus intensity-modulated radiotherapy (IMRT) boosts after breast-conserving surgery in nodal-negative female patients, including two subgroups of 76 patients in each, respectively. Radiation therapy was applied between January 2002 and March 2015, and patients were followed-up with until 11 March 2022 for local control rate as primary endpoint. Secondary endpoints represented overall survival, progression-free survival, cosmetic results, and the incidence of acute and late toxicity. The evaluation of acute and chronic adverse effects was based on the Common Terminology Criteria for Adverse Events v4.0 (CTCAE) for acute toxicity and the LENT SOMA scale for late toxicity. Adverse effects within 90 days after radiotherapy were classified as acute, events occurring >90 days after end of RT were categorized as chronic toxicities. This means that, in the IORT-subgroup, adverse events such as skin reactions, wound healing disorders, and pain were defined as acute when occurring in the period of <90 days after surgery including intraoperative radiotherapy. The cosmetic result was assessed during radiotherapy series by means of two scores, one for subjective evaluation and one for objective evaluation. The survey of the objective cosmetic result was performed by a physician during radiotherapy series. The subjective cosmetic result was then surveyed again during follow-up after radiation treatment.

Overall survival was defined as the period between surgery and any cause of death or the last follow-up. Progression-free survival described the period between surgery until recurrence or progression of the disease.

### 2.1. Patients

This study included *n* = 152 patients, 76 patients in each subgroup. Inclusion criteria were invasive breast carcinoma, nodal status negative (N0), curative treatment concept without evidence of distant metastasis (M0), breast-conserving surgery, stage I–II, tumor diameter ≤ 3 cm, distance from the skin ≥ 5 mm, and multifocality or multicentricity disease (exception for neighboring tumor foci). Patients with non-invasive breast carcinoma, palliative concept including distant metastasis, mastectomy, stage III–IV, tumor diameter > 3 cm, and distance from the skin < 5 mm were excluded from the study.

### 2.2. Surgery and Radiation Treatment

In the IORT subgroup, the boost was applied during surgical treatment after complete resection of the tumor as a single dose of 9 Gy with electron energies between 6 and 12 MeV. A 90% reference isodose covering the planned target volume was reached. For the application of the IORT boost, tube sizes between 4 and 5 cm in diameter were used. A large focus was placed on lung sparing. Therefore, to not overstep the dose limit of 3 Gy delivered to the lung, additional shielding of the lung was induced with a 2 cm-thick lead plate placed between breast tissue and musculus pectoralis major.

To align the tube precisely to the lead plate, a magnetic system on the lead plate was used. An additional small ball in the end of the tube with a magnetic pyramid on top of it helped to position the tube in a vertical position to the lead plate. Afterwards, all patients were given a whole breast irradiation (WBI) with 50.4/50 Gy in 1.8–2 Gy per fraction [24].

With the continuing development of IMRT-technologies, it was possible to administer the boost percutaneously, and simultaneously integrated into the tumor bed (representing the IMRT-subgroup). All patients received a single dose boost of 0.3 Gy in 5 fractions per week up to 8.4 Gy total boost dose with electron energies between 6 and 15 MeV. Thereby, a cumulative dose of 58.8 Gy in the boost area was applied.

### 2.3. Follow-Up

This study addressed follow-ups until 11 March 2022. A questionnaire including the issues tumor control, recurrences, chronic toxicities based on the CTCAE, and cosmetic results were personally evaluated with each patient.

### 2.4. Statistic Methods

For the analysis of patients data, the software SPSS (IBM SPSS Statistics, IBM Corporation, Armonk, NY, USA, Version 27.0.0.0) was used. Using the Kaplan–Meier method, the overall survival for each subgroup was calculated. The difference between the IORT and IMRT subgroups was evaluated by the log-rank test. Univariate and multivariate Cox regression were used to compare the progression-free survival of the subgroups and to check for influencing factors. With the Fisher test, the frequency of acute toxicities distributed among the subgroups was analyzed. *p* values of ≤0.05 were classified as statistically significant.

## 3. Results

In this study, 152 female patients were examined for pathologic tumor size, histology, multifocality, grading, resection status, secondary resection or mastectomy, hormonal status, adjuvant therapy, and acute and chronic toxicities, and were divided into two subgroups with 76 patients each. The distribution of tumor histology in the intraoperative radiotherapy (IORT) boost subgroup and in the intensity-modulated (IMRT) boost subgroup was almost equal; 80.3% of patients in the IORT subgroup had invasive ductal breast carcinoma (IDC)and 77.6% had invasive breast carcinoma of no special type (NST) based on the new nomenclature in the IMRT subgroup. The parameters histology, pathological tumor stage, and neoadjuvant chemotherapy resulted in *p*-values < 0.05, which shows a significant correlation between the parameters and the subgroup. In the IORT subgroup, 20/76 patients received chemotherapy. In 18/76 cases from the IMRT subgroup, chemotherapy was given, and out of them, in 9/18 patients, it was given as neoadjuvant treatment. The neodjuvant application of chemotherapy was introduced later and, therefore, was only used in the IMRT subgroup. After neoadjuvant chemotherapy in the IMRT subgroup, a downstaging of the tumor was revealed in 7/9 patients. In one case, the neoadjuvant chemotherapy led to downsizing without any influence on the stage, whereas, in one case, upsizing within stage I occurred. A more detailed evaluation can be found in Table 1. Due to the inclusion criteria, all patients showed negative nodal status (N0). Only two patients of 76 from the IORT subgroup and no patients from the IMRT subgroup showed a multifocal tumor. Almost all situations were similar for resection status: three patients showed with R1, while in the IMRT subgroup, all patients showed R0. The distribution of hormone receptor status can be found in Table 2.

No patient underwent secondary mastectomy; in 11 patients in the IORT subgroup and in 20 patients in the IMRT subgroup, secondary resection was performed. In two cases in the IORT subgroup, additional radiotherapy of ipsilateral supra-/infraclavicular lymph nodes was applied to the lymph drainage pathways, despite the patients’ nodal status being negative. In these two IORT cases 7/10 lymph nodes without tumor cell involvement were resected and, based on the guidelines in force in the years 2002–2003, the additional radiotherapy of lymph nodes took place as a precautionary measure.

Within a median follow-up time of 7.92 years (range 0.17–20 years), one patient out of the IMRT subgroup developed a local recurrence with cutaneous, pulmonary, lymphonodal, and osseous distant metastasis one year after surgery, resulting in a *p* value of 0.363. In three other cases, osseous, hepatic, pulmonary, and cerebral distant metastasis appeared (one IMRT, two IORT). The *p* value determined with the chi-square-test was 0.604. The one case with a local recurrence raises the speculation of an in-field recurrence since the recurrence appeared shortly after radiotherapy. The patient died of tumor-related causes despite several lines of systemic therapy; the same is true for the other two cases of distant metastasis. One patient is still alive with osseous metastasis since the progression of the disease could be controlled under radiotherapy, and Palbociclib and antihormonal therapy. The other patients with metastasis died. Secondary malignancies developed in 16 patients (11 IORT, 5 IMRT), five in ipsilateral breast, four in contralateral breast, three in lung, one intestinal, two endometrium carcinoma, and one fallopian tube carcinoma. None of the cases appear to be therapy-induced. The Chi-square-test resulted in *p* = 0.125. The aforementioned patients were treated with different approaches of systemic therapies: six patients received radiotherapy of the breast region, two of them also received radiotherapy of ipsilateral supra-/infraclavicular lymph nodes, five patients received breast-conserving surgery, and five patients received mastectomy. In three cases, chemotherapy was performed, and seven patients received endocrine therapy, while antibody therapy was used in two cases. Apart from the breast, second cancers also occurred in other organs; therefore, further surgery was performed in three, chemotherapy in two, and radiotherapy in three cases, respectively. In four cases, further therapies are unknown. Apart from three patients (one endometrium carcinoma, one fallopian tube carcinoma, and one lung carcinoma), a curative concept was achieved. Although a suspicious focus was discovered in one patient, nothing could be found out about the further course of the disease due to lack of information.

**Figure 1:**
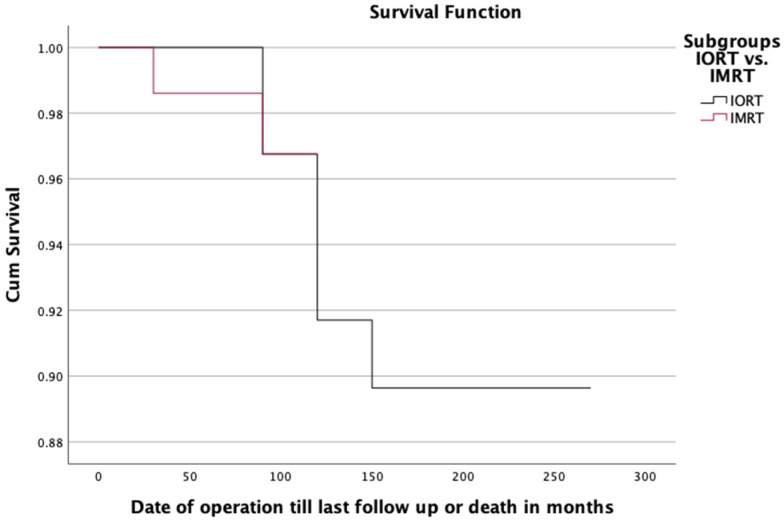
Kaplan–Meier curve comparing the overall survival of IORT and IMRT subgroups. Cumulative survival rate showing how many clients have not had an event occur by a point in time.

During the follow-up period, eight patients died (six IORT; two IMRT; 5.26%). The overall survival (OS) after five years was 100% (IC95% 17.95–19.72 years) in the IORT subgroup and 98.5% in the IMRT subgroup (IC95% 8.27–8.65 years) (Figure 1). A *p* value of 0.917 in the Log-rank test indicates that there was no significant difference between both subgroups, including competing risk. Three patients from the entire cohort (two IORT, one IMRT) died of tumor-related causes (in relation to primary tumor). With a *p* value of 0.310 and a Hazard Ratio of 67.233, no statistical significance could be shown. Three patients (two IORT, one IMRT) died of causes related to a secondary tumor. With a *p* value of 0.640 and a Hazard Ratio of 0.568, no statistical significance can be proved. No patient died of therapy-related causes. The third cause of death was defined as a non-tumor-associated death, from which two patients died (only IORT). For progression-free survival (PFS), when comparing the two subgroups (IORT vs. IMRT), no predictive factor could be found (Table 3). According to the Hazard Ratio found via univariate analysis, only the administration of chemotherapy, antihormonal therapy, and radiotherapy of ipsilateral supra-/infraclavicular lymph nodes seemed to favor progression-free survival.

As acute toxicity, two patients equally distributed over both subgroups showed wound healing disorders. Seroma formation was revealed in five cases (four in the IORT subgroup, one in the IMRT subgroup). A hematoma occurred in four cases (three IORT, one IMRT). In the IMRT subgroup, 19 patients showed a low-grade breast edema after radiotherapy, while in the IORT subgroup, only 5 breast edemas occurred. After radiotherapy, 60 patients (28 IORT, 32 IMRT) developed a maximum first-degree skin reaction, 17 patients (4 IORT, 13 IMRT) developed a second-degree skin reaction, and 7 patients showed a third-degree skin reaction (all from IMRT subgroup). Nausea (one IORT, five IMRT), hot flashes (two IORT, five IMRT), overheating of breast skin (three IORT, one IMRT), and feeling of pressure (four patients from IMRT-subgroup) rarely occurred. Serious consequences for lung or heart were not observed in any patient.

The Fisher test, which was used to determine the relationship between the type of boost administration and the occurrence of acute toxicities, resulted in a *p* value < 0.001 after radiotherapy. showing that the variables are dependent and that patients in the IMRT subgroup displayed more acute side effects after irradiation than IORT patients: 58/76 IMRT patients showed acute side effects while, after IORT, acute toxicities were noticed in only 10/76 cases. After IORT and completed whole breast irradiation in the IORT subgroup, 33/76 patients showed acute toxicities and the gap between the subgroups became smaller.

In the follow-up evaluation, 118 (56 IORT, 62 IMRT) out of 152 patients participated and the chronic toxicities were surveyed based on the LENT SOMA scale, which provides a classification of toxicity into subjective and objective, and into scoring of grades 1–4. The objective assessment was requested from the patients and compared with the file entries of the 118 patients from the last examination. In Table A1, the assessments of subjective and objective breast-, heart-, and lung-related late toxicities are listed. *p* values calculated with the Chi-square test showed no significant associations between the subgroup and the respective adverse event, similar to the Fisher test, which had a *p* value of 1.000. In alignment with the record entries of the most recent classification of chronic toxicities by physicians, for 29/52 IORT and 52/62 IMRT patients, objective chronic adverse events were evaluated. In the IORT subgroup, six patients showed grade one hyperpigmentation of skin, in five cases teleangiectasis grade one occurred, and four patients developed a first-grade retraction or atrophy of the irradiated breast. Induration and ulcer (grade one) appeared in one case. Grade one fibrosis showed up in 11 cases, and a second-grade fibrosis was seen in 1 case. Lymphedema in the treated region of the breast was observed with a grade one reaction in four cases and a grade two reaction in one case. A grade one edema appeared in two cases. Two patients developed a lymphedema in the ipsilateral arm: one patient developed a first-grade lymphedema and one patient a second-grade lymphedema. Two patients showed a grade one reaction of lung in the form of an irritating cough. Eleven patients of the IORT subgroup did not show any late effects.

In the IMRT subgroup, 13 patients described first-grade skin reactions. One patient showed a grade two hyperpigmentation, one patient an induration, and one showed patient retraction or atrophy (grade one) of the breast. Teleangiectasis occurred with a first-grade reaction in three cases. One patient developed a second grade teleangiectasis. Eight patients pointed out a fibrosis (grade one) and one patient a grade two fibrosis. Lymphedema in the region of the arm occurred in three cases (two grade one and one grade two). Three patients showed a grade one lymphedema and three patients a grade two lymphedema in the breast region. A radiogenic reaction in the region of the lung occurred in four cases (three grade one, one grade three). Two patients developed a grade one reaction in the region of the heart in the form of a cardiac arrhythmia. In total, 28 patients did not show any chronic toxicities at the most recent examination.

Other late toxicities reported by patients were the feeling of faster fatigue since the primary treatment, chronic pulling in the chest, movement restrictions (in the area of arms or ribs), and a numb feeling in the irradiated breast.

**Figure 2:**
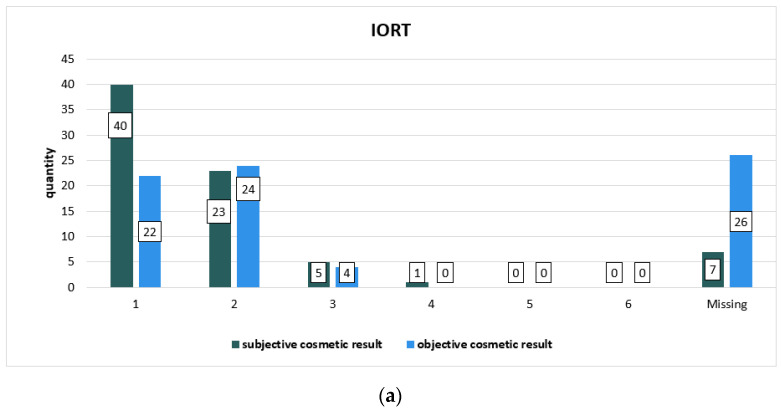

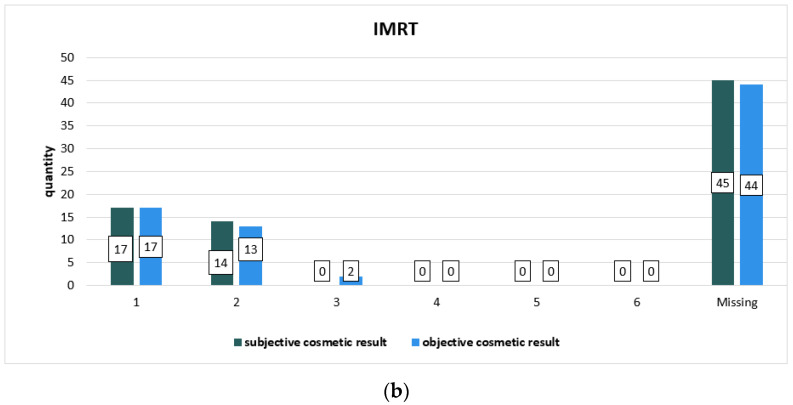
Graphic representation of the comparisons of subjective cosmetic results evaluated by patients during radiotherapy series and objective cosmetic results evaluated by physicians during radiotherapy series divided into subgroups: (**a**) for IORT and (**b**) for IMRT subgroups. The columns represent the number of cases.

**Figure 3:**
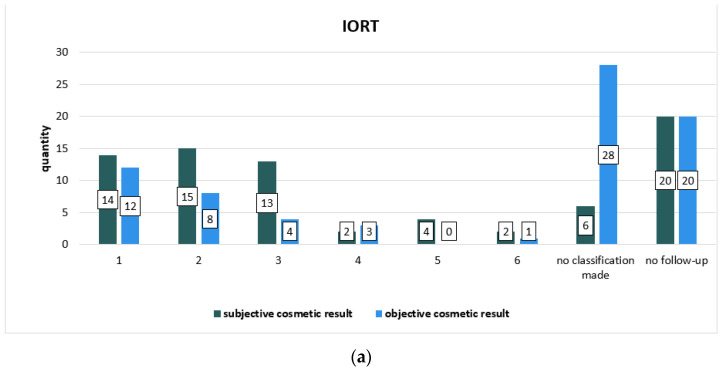

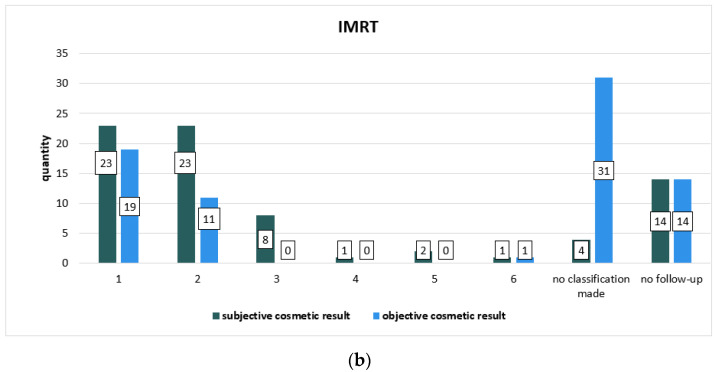
Graphic representation of the comparison of the subjective cosmetic result collected during follow-up to the objective cosmetic result collected during follow-up classified by the treating physician, one graph for (**a**) IORT subgroup and one for (**b**) IMRT subgroup. The columns represent the number of cases.

The cosmetic result was collected at two times during radiation treatment and follow-up: subjective by patients and objective by physicians during radiotherapy series and subjective by patients and objective by treating physician at follow-up visits. Table 4 focuses on the subjective cosmetic result during radiation, Table 5 sums up the objective cosmetic result evaluated by physicians during the radiotherapy series, and Figure 2 compares the subjective cosmetic result with the objective cosmetic result evaluated during the radiotherapy series. Table 6 and Table 7 and Figure 3 show the results of the follow-up on the subjective cosmetic result evaluated by patients in comparison to the objective cosmetic result evaluated by the physicians providing treatment during follow-up. The Chi-square test showed no statistically significant differences in the two subgroups in terms of the subjective and objective cosmetic result. There were no significant differences between the patients’ own assessment and that of their physician.

## 4. Discussion

The current guidelines for the continuing treatment of invasive breast cancer patients undergoing conservative breast surgery include an adjuvant radiotherapy administered as a hypofractionated (e.g., 40.05 Gy in daily doses of 2.67 Gy) or conventionally fractionated (50.4/50 Gy in daily doses of 1.8–2 Gy) treatment concept [1,25]. Evidenced by further studies, applying a boost (defined as an extra radiation dose to the tumor bed) improves local control rates, since “(…) (i) local recurrence occurs mostly at the site of the primary tumor because remaining microscopic tumor cells are most likely situated here; and (ii) radiation can eliminate these causative microscopic tumor cells [26].” Boosts can be applied as a sequential boost with electrons, with photons, or as interstitial brachytherapy [1]. Other techniques discussed here are intraoperative radiotherapy (IORT), either with KV or electrons and a simultaneous integrated boost (SIB) during adjuvant whole breast irradiation. The target group for boost application turned out to be patients </=50 years and >50 years with established risk factors for recurrences (e.g., triple negative breast carcinoma, G3, Her2-positive, >T1) since, in younger patients, a higher recurrence rate has been recognized [1,27].

In the phase III randomized trial by the European Organisation for Research and Treatment of Cancer (EORTC) in which the efficacy of a 16 Gy boost compared to no boost application in terms of overall survival and local control in a 20-year-follow-up was proven, but also the associated moderate fibrosis was underlined, it turned out that using a boost on the tumor bed does not lead to significant survival benefits [28]. This observation was also substantiated in a randomized clinical trial by Clark et al., in which a comparison of no radiation to radiation with boost application led to a reduction in local recurrences; however, no statistically significant effect in mortality was shown [4,5]. Despite that a boost decreases the rate of ipsilateral breast recurrences regardless of age, it was observed that, with increasing age, “the absolute gain in local control decreased (in proportion to the absolute risk of relapse)” [28]. It must not be disregarded that these positive results are accompanied by a higher risk of fibrosis.

The cosmetic result was examined as a part of the systematic review of tumor bed boosts by Kindts et al., where a boost application was compared with no boost application. A better subjective result was demonstrated in patients in the no-boost group, and objective results raised by physicians did not differ between the no-boost and boost subgroup [26].

The cosmetic outcome was also a secondary endpoint in the Young Boost Trial by Brouwers et al., in which patients received a boost dose randomized between 26 Gy and 16 Gy to analyze the factors that influence poor cosmetic outcome. The cosmetic outcome as a secondary endpoint was evaluated at baseline (meaning after breast conserving surgery and before radiotherapy), one and four years after treatment. Influencing factors for worse cosmetic results turned out to be the use of photons, a high boost dose, and adjuvant chemotherapy. A large boost volume and whether the cosmetic outcome was already poor prior to radiotherapy also represent predictive factors for a poor cosmetic result [29].

A collaborative analysis has been performed by seven European International Society of Intraoperative Radiation Therapy (ISIORT) member institutions with 1109 unselected patients, which analyzed a long-term evaluation of a 10 Gy intraoperative radiotherapy with electrons (IOERT) boost after breast conserving therapy and prior to 50–54 Gy whole breast irradiation. It showed that, since the skin as a tissue at risk is spared and, as small treatment volumes are allowed in the IOERT-boost technique, the conclusion can be drawn that IOERT leads to lower skin reactions and as well is advantageous in terms of precision. In this context, local control rates and patient comfort were also improved, since the IOERT boost shortens treatment times compared to external boost [12]. IOERT combined with a hypofractionated external beam WBI (HWBI) was the topic of the HIOB trial, a prospective multicenter trial of the ISIORT published in 2020. It turned out that IOERT with HWBI leads to excellent overall tissue tolerance and T-size, tube diameter, age, and fibrosis are important influencing factors for poor cosmetic outcome [30].

A single-institution phase III randomized study investigated by Ciabattoni et al. compared intraoperative radiotherapy with electrons of 10 Gy with external beam radiotherapy (EBRT) with 5 × 2 Gy [22]. Compared with the present study, in the study by Ciabattoni et al., external boost was applied sequentially and with electrons. This study supports the hypothesis that IOERT leads to better cosmetic outcomes, as both the patients’ and physicians’ categorization resulted in significantly better outcomes, with the biggest difference in the evaluation at the end of EBRT with *p* values of 0.006 (objective) and 0.0004 (subjective) [22]. In addition, it has been observed that recurrences occurred significantly earlier in the EBRT subgroup after a mean time of about 55.2 months compared to >100 months in the IOERT subgroup; however, even with a tendency towards a better result in the IOERT arm, the overall survival and local control did not differ significantly between the two subgroups [22].

A sequential intervention study by Reitsamer et al. treated 188 of 378 patients with a postoperative electron boost of 12 Gy and 190 of the 378 patients with an intraoperative electron radiotherapy of 9 Gy. The authors verified the hypothesis that using IORT might lower the risk of topographic and temporal misses [31], which could be more common in external boost techniques because dose-limiting tissue is excluded, and the direct visualization during surgery and hence a more precise definition of the target volume is made possible. This consideration is supported in the study by an improved local control rate and leads to the conclusion that intraoperative boost is better than conventional postoperative boost [23].

The aim of the present study was the comparison of a boost applied as an intraoperative radiotherapy (IORT) with a boost applied as an intensity-modulated radiotherapy (IMRT). The IMRT boost was applied simultaneously integrated, as it is known for the improvement of target dose conformality and reduction in normal tissue doses. Simultaneously-integrated boosts, such as IMRT have been shown to be advantageous over 3D-conformal radiotherapy, especially in left-sided breast cancer [32,33].

Comparing the two subgroups regarding the study parameters, the distribution of neoadjuvant chemotherapy was indeed different. Nevertheless, the comparability of the two subgroups is confirmed, as the proportion of patients with additional chemotherapy is comparable with 20 IORT patients and 18 IMRT patients (*p* value of 0.708). Against the background of the development of neoadjuvant chemotherapy concepts, these were used only in the IMRT collective.

The rate of recurrences was higher in the IMRT subgroup since only one patient from the IMRT and none out of the IORT subgroup developed a local recurrence. The rate of ipsilateral secondary breast tumors was higher in the IORT subgroup with three patients, versus one patient in the IMRT subgroup, and with equal distribution of contralateral secondary tumors. Secondary tumors that have occurred in other organs showed an almost equal distribution. Statistically, no significance was revealed, and the Cox proportional hazard regression model which was used to analyze progression-free survival showed no differences between the IORT and IMRT subgroups.

This study showed statistically significant lower acute skin reactions in the IORT subgroup (after IORT and whole breast irradiation) than in the IMRT subgroup, with a *p*-value of <0.001. However, regarding late toxicities, between the IORT and IMRT group, no significant differences in any case could be found and, in this respect, no technique can be considered superior to the other.

Including competing risks, no differences in the present study for overall survival were found. This study proves that the mode of boost administration has no effect on mortality. The results are not decisive due to the small group of events, as a total of eight patients died, of which two patients died from non-tumor-associated causes. The Hazard Ratio, as an effect measure, showed no difference between both subgroups (IORT and IMRT); therefore, even if there is a difference visible in the data set, it cannot be ruled out that it is only a random difference between the subgroups IORT and IMRT.

In this study, no statistically significant difference could be found between subjective and objective cosmetic results whether evaluated by the patient herself or by the treating physician.

Lastly, there were some limitations to this study. In the IMRT subgroup, no patient was <41, whereas, in the IORT subgroup, the youngest patient was 29 years old. As known from previous studies, age is an important influencing factor on the local recurrence rate: the younger the patient, the higher the recurrence rate [12]. In this study, the average age was 53.50 years in the IORT and 67 years in the IMRT subgroup; however, age as a negative influencing factor could not be substantiated in this study due to the absence of <41-year-old patients in the IMRT subgroup, which removes the possibility of comparing the two subgroups with regard to younger patients.

## 5. Conclusions

The comparison of intraoperative radiotherapy (IORT) boost and intensity-modulated radiotherapy (IMRT) boost showed no significant difference in overall survival, local control rate, progression-free survival, cosmesis, or late toxicity. The lack of significant difference in overall survival leads to the conclusion that the technique of boost administration has no effect on mortality, just as the boost administration itself, which has already been described in previous comparative studies. The results of the cosmetic outcome did not differ significantly between the classifications of the patients themselves and those of the treating physicians, nor between the classifications between the two subgroups, IORT and IMRT. No risk factor turned out to be significant for progression-free survival. It seems that the administration of chemotherapy, antihormonal therapy, and radiotherapy of ipsilateral supra-/infraclavicular lymph nodes tends to favor progression-free survival. Acute toxicity seems to be less after IORT for the time being; however, after the completion of adjuvant radiotherapy, this difference seems to even out. Hence, it cannot be determined whether one of the two boost application techniques leads to better results than the other with respect to the analyzed parameters.

## Figures and Tables

**Table 1:** Comparison of subgroups on different study parameters. *p* values were evaluated with chi-square test.

	IORT-Boost	IMRT-Boost	Primary Tumor Stage	Postoperative Tumor Stage	*p* Value
**Group size**	76	76			
**Age average (range) [y]**	53.50 (29–68)	67.00 (41–90)			
**Histology**					0.691
IDC/NST ***	61 (80.3%) (IDC)	59 (77.6%) (NST)			
ILC	4 (5.3%)	12 (15.8%)			
mixed	5 (6.6%)	1 (1.3%)			
other (tubular, mucinous, medullary)	4 (5.3%)	4 (5.3%)			
atypical medullary	2 (2.6%)	0			
**Primary Tumor stage**					0.006 **
pT1	68 (89.5%)	53 (69.7%)			
pT2	7 (9.2%)	14 (18.4%)			
pT3–4	0	0			
cT1b	0	2 (2.6%)			
cT2	0	7 (9.2%)			
(m)pT1c	1 (1.3%)	0			
∑ T1–2	76	76			
**Grading**					0.348
G1	22 (28.9%)	18 (23.7%)			
G2	39 (51.3%)	36 (47.4%)			
G3	14 (18.4%)	22 (28.9%)			
missing	1 (1.3%)	0			
**Chemotherapy**					0.708
yes	20 (26.3%)	18 (23.7%)			
no	56 (73.7%)	58 (76.3%)			
**Neoadjuvant Chemotherapy**					0.002 **
yes	0	9 (11.8%)			
			cT2	ypT0	
			cT2	ypT0	
			cT2	ypT0	
			cT2	ypT1	
			cT1b	ypT1b	
			cT2	ypt1c	
			cT2	ypTis	
			cT2	ypT0	
			cT1b	ypT1c	
no	76 (100%)	67 (88.2%)			
**Endocrine therapy**					0.182
yes	61 (80.3%)	67 (88.2%)			
no	15 (19.7%)	9 (11.8%)			

Abbreviations: y = years; IDC = invasive ductal breast carcinoma; NST = invasive breast carcinoma of no special type; ILC = invasive lobular breast carcinoma; m = multifocal. ** = considered as significant. *** NST corresponds to the former IDC subtype.

**Table 2:** Distribution of receptor status in the cohort.

Receptor Status	Frequency	Percent (%)
ER+, PR+, Her2 neu 0	9	5.9
ER+, PR+, c-erbB2 2+	1	0.7
ER+, PR−, c-erbB2−	5	3.3
ER−, PR+, c-erbB2−	1	0.7
ER−, PR−, c-erbB2 3+	1	0.7
ER−, PR−	2	1.3
ER+, PR−	1	0.7
ER+, PR+	1	0.7
ER−, PR−, Her2 neu −	4	2.6
ER−, PR−, Her2 neu +	1	0.7
ER+, PR+, Her2 neu 1+	9	5.9
ER−, PR−, Her2 neu 3+	5	3.3
ER+, PR−, Her2 neu 2+	1	0.7
ER+, PR+, Her2 neu 3+	2	1.3
ER+, PR+, Her2 neu −	78	51.3
ER+, PR−, Her2 neu 3+	1	0.7
ER+, PR−, Her2 neu 1+	4	2.6
ER+, PR−, Her2 neu −	9	5.9
ER+, PR+, Her2 neu 2+	5	3.3
ER+, PR+, c-erbB2+	4	2.6
ER+, PR−, c-erbB2+	1	0.7
ER−, PR−, c-erbB2−	6	3.9
ER−, PR+, c-erbB2−	1	0.7
∑	152	100

**Table 3:** Univariate and multivariate Cox proportional hazard regression model with Hazard ratio (HR) and 95% confidence intervals (95% CI) analyzing progression-free survival while comparing subgroups.

Influencing Factor	Univariate *p* Value/HR/Cl 95%	Multivariate *p* Value	HR/Cl 95%
age	0.764/1.009/0.949–1.074	0.592	0.992/0.927–1.062
grading	0.122/2.067/0.805–5.311		3.066/0.953–9.866
HR-status	0.690/1.025/0.905–1.161		0.999/0.882–1.132
pathological T stage	0.912/0.967/0.540–1.731		1.545/0.581–4.111
histology	0.311/1.378/0.778–2.441		1.563/0.739–3.081
chemotherapy	0.849/0.860/0.178–4.150		0.300/0.041–2.210
neoadjuvant chemotherapy	0.297/3.775/0.440–32.374		17.295/0.160–1874.099
antihormonal therapy	0.730/0.752/0.155–3.638		1.313/0.184–9.354
resection status	0.586/1.582/0.369–6.793		0.868/0.116–6.490
LAW	0.525/0.048/0.000–12,334,080.6		0.000/0.000

**Table 4:** Subjective cosmetic result evaluated during radiotherapy series, scale equal to school grades (1: very good, 6: poor).

Subjective Cosmetic Result	IORT	IMRT	*p* Value (Chi-Square-Test)
1	40 (52.6%)	17 (22.4%)	0.315
2	23 (30.3%)	14 (18.4%)	
3	5 (6.6%)	0	
4	1 (1.3%)	0	
5	0	0	
6	0	0	
Missing	7 (9.2%)	45 (59.2 %)	

**Table 5:** Objective cosmetic result evaluated by physicians during radiotherapy series, scale equal to school grades (1: very good,.6: poor).

Objective Cosmetic Result	IORT	IMRT	*p* Value (Chi-Square-Test)
1	22 (28.9%)	17 (22.4%)	0.719
2	24 (31.6%)	13 (17.1%)	
3	4 (5.3%)	2 (2.6%)	
4	0	0	
5	0	0	
6	0	0	
Missing	26 (34.2%)	44 (57.9%)	

**Table 6:** Subjective cosmetic result evaluated during follow-up, scale equal to school grades (1: very good, 6: poor).

Subjective Cosmetic Result	IORT	IMRT	*p* Value (Chi-Square-Test)
1	14 (18.4%)	23 (30.3%)	0.345
2	15 (19.7%)	23 (30.3%)	
3	13 (17.1%)	8 (10.5%)	
4	2 (2.6%)	1 (1.3%)	
5	4 (5.3%)	2 (2.6%)	
6	2 (2.6%)	1 (1.3%)	
No classification made	6 (7.9%)	4 (5.3%)	
No follow-up	20 (26.3%)	14 (18.4%)	

**Table 7:** Objective cosmetic result evaluated during follow-up, assessment of the physician providing treatment, scale equal to school grades (1: very good, 6: poor).

Objective Cosmetic Result	IORT	IMRT	*p* Value (Chi-Square-Test)
1	12 (15.8%)	19 (25.0%)	0.114
2	8 (10.5%)	11 (14.5%)	
3	4 (5.3%)	0	
4	3 (3.9%)	0	
5	0	0	
6	1 (1.3%)	1 (1.3%)	
No classification made	28 (36.8%)	31 (40.8%)	
No follow-up	20 (26.3%)	14 (18.4%)	

## Data Availability

Data is contained within the article.

## Appendix A

**Table A1:** Chronic toxicities based on LENT SOMA scale collected during follow-up according to subgroups. Classification of heart-, lung- and breast-related late toxicities in subjective (self-assessed by patients) and objective events (assessment of the physician providing treatment ) in grades 1–4.

	Adverse Event	IORT	IMRT	*p* Value (Chi-Square Test)
**BREAST** **SUBJECTIVE**	PAIN	23 (30.3%)	29 (38.2%)	0.355
	Unknown	-	-	
	Grade			
	1	18	23	
	2	3	4	
	3	1	2	
	4	1	0	
	Unknown	-	-	
	Relation to RT			
	Yes	21	28	
	Unknown	-	-	
**OBJECTIVE**	BREAST EDEMA	5 (6.6%)	5 (6.6%)	0.427
	Unknown	-	-	
	Grade			
	1	2	2	
	2	3	3	
	Unknown	-	-	
	Relation to RT			
	Yes	3	5	
	Unknown	-	-	
	FIBROSIS	31 (40.8%)	23 (30.3%)	0.135
	Unknown	-	-	
	Grade			
	1	12	11	
	2	16	9	
	3	4	3	
	Unknown	-	-	
	Relation to RT			
	Yes	27	23	
	Unknown	-	-	
	TELEANGIECTASIS	2 (2.6%)	2 (2.6%)	0.503
	Unknown	-	-	
	Grade			
	1	1	2	
	2	1	0	
	Unknown	-	-	
	Relation to RT			
	Yes	2	2	
	Unknown	-	-	
	LYMPHEDEMA (ARM)	13 (17.1%)	10 (13.2%)	0.428
	Unknown	-	-	
	Grade			
	1	8	8	
	2	3	0	
	Unknown	2	2	
	Relation to RT			
	Yes	11	10	
	Unknown	1	0	
	RETRACTION/			0.300
	ATROPHY	11 (14.5%)	10 (13.2%)	
	Unknown	-	-	
	Grade			
	1	8	9	
	2	0	1	
	3	2	0	
	4	1	0	
	Unknown	-	-	
	Relation to RT			
	Yes	7	9	
	Unknown	2	0	
	ULCER	2 (2.6%)	0	0.290
	Unknown	1	1	
	Grade			
	Unknown	2	1	
	Relation to RT			
	Yes	1	-	
	Unknown	-	1	
**HEART** **SUBJECTIVE**	ANGINA PECTORIS	9 (11.8%)	7 (9.2%)	0.537
	Unknown	-	-	
	Grade			
	1	4	5	
	2	1	2	
	3	3	0	
	4	1	0	
	Unknown	-	-	
	Relation to RT			
	Yes	2	1	
	Unknown	3	2	
	PERICARDIAL PAIN	7 (9.2%)	3 (3.9%)	0.658
	Unknown	3	5	
	Grade			
	1	6	7	
	2	2	1	
	Unknown	-	-	
	Relation to RT			
	Yes	2	3	
	Unknown	2	3	
	PALPITATION	23 (30.3%)	30 (39.5%)	0.184
	Unknown	-	1	
	Grade			
	1	18	15	
	2	4	10	
	3	1	4	
	4	0	2	
	Unknown	-	-	
	Relation to RT			
	Yes	5	3	
	Unknown	4	11	
	DYSPNEA	29 (38.2%)	32 (42.1%)	0.409
	Unknown	-	1	
	Grade			
	1	14	24	
	2	13	7	
	3	2	1	
	Unknown	-	-	
	Relation to RT			
	Yes	8	6	
	Unknown	2	5	
	PEDAL EDEMA	11 (14.5%)	21 (27.6%)	0.165
	Unknown	-	-	
	Grade			
	1	6	13	
	2	3	6	
	3	2	2	
	Unknown	-	-	
	Relation to RT			
	Yes	0	3	
	Unknown	-	1	
**OBJECTIVE**	PEDAL EDEMA	11 (14.5%)	21 (27.6%)	0.165
	Unknown	-	-	
	Grade			
	1	1	4	
	2	0	5	
	3	0	3	
	4	0	2	
	Unknown	10	7	
	Relation to RT			
	Yes	0	3	
	Unknown	-	1	
	CARDIOMEGALY	0	5 (6.6%)	0.202
	Unknown	1	1	
	Grade			
	1	0	1	
	2	0	2	
	3	0	1	
	Unknown	1	1	
	Relation to RT			
	Yes	0	2	
	Unknown	-	-	
	CARDIAC DYSRHTYTHMIA	4 (5.3%)	18 (23.7%)	0.711
	Unknown	1	-	
	Grade			
	1	1	7	
	2	1	3	
	3	1	1	
	4	1	2	
	Unknown	-	5	
	Relation to RT			
	Yes	1	1	
	Unknown	1	1	
	MYOCARIDAL CHF	3 (3.9%)	4 (5.3%)	0.214
	Unknown	1	-	
	Grade			
	1	0	1	
	Unknown	2	3	
	Relation to RT			
	Yes	0	1	
	Unknown	-	-	
	MYOCARDIAL			0.290
	ISCHEMIA	1 (1.3%)	2 (2.6%)	
	Unknown	2	2	
	Grade			
	1	0	0	
	2	0	0	
	3	0	0	
	4	1	1	
	Unknown	-	1	
	Relation to RT			
	Yes	0	0	
	Unknown	1	-	
	PERICARDIAL			0.243
	DISEASE	0	0	
	Unknown	-	-	
	Grade			
	Unknown	-	-	
	Relation to RT			
	Yes	-	-	
	Unknown	-	-	
**LUNG** **SUBJECTIVE**	COUGH	19 (25%)	29 (38.2%)	0.383
	Unknown	-	-	
	Grade			
	1	8	11	
	2	4	7	
	3	5	6	
	4	3	5	
	Unknown	-	-	
	Relation to RT			
	Yes	6	12	
	Unknown	5	5	
	DYSPNEA	29 (38.2%)	32 (42.1%)	0.418
	Unknown	-	1	
	Grade			
	1	15	24	
	2	13	7	
	3	2	1	
	Unknown	-	-	
	Relation to RT			
	Yes	9	6	
	Unknown	2	4	
	CHEST PAIN	4 (5.3%)	7 (9.2%)	0.427
	Unknown	-	-	
	Grade			
	1	1	5	
	2	1	2	
	3	2	0	
	Unknown	-	-	
	Relation to RT			
	Yes	3	5	
	Unknown	-	-	
**OBJECTIVE**	PULMONARY			0.230
	FIBROSIS	2 (2.6%)	4 (5.3%)	
	Unknown	2	2	
	Grade			
	1	1	2	
	2	0	1	
	Unknown	1	1	
	Relation to RT			
	Yes	1	4	
	Unknown	-	-	
	LUNG FUNCTION	16 (21.1%)	19 (25%)	0.448
	Unknown	1	-	
	Grade			
	1	10	14	
	2	4	2	
	3	0	1	
	Unknown	2	2	
	Relation to RT			
	Yes	6	5	
	Unknown	-	-	
